# Towards Modernization of the Formulation of the Traditional Uighur Medicine Herbal Preparation *Abnormal Savda Munziq*

**DOI:** 10.1155/2012/863101

**Published:** 2011-08-07

**Authors:** Murat Kizaibek, Ruxandra Popescu, Sonja Prinz, Halmurat Upur, Judith Singhuber, Martin Zehl, Brigitte Kopp

**Affiliations:** ^1^Faculty of Traditional Uighur Medicine, Xinjiang Medical University, Urumqi 830011, China; ^2^Department of Pharmacognosy, University of Vienna, Althanstraße 14, 1090 Vienna, Austria

## Abstract

*Abnormal Savda Munziq* (ASMq) is a herbal preparation used in Traditional Uighur Medicine for the treatment and prevention of diabetes, cardiovascular diseases, chronic asthma and cancer. The recommended dose of this decoction for cancer patients is 500 mL administered orally three times a day. Our approach aimed at reducing the high amount of fluid intake required by fractionation of ASMq guided by the antiproliferative activity on HL-60 cells. The fractionation of ASMq resulted in the preparation of an active extract, Extr-4. Using solid phase extraction, Extr-4 was further fractionated into five fractions (SPE-0, SPE-20, SPE-40, SPE-60 and SPE-80), with SPE-40 showing the strongest antiproliferative activity. Caffeic acid, rutin, isoquercitrin, isorhamnetin 3-O-rutinoside, apigenin 7-O-glucoside, rosmarinic acid, luteolin and formononetin were identified in Extr-4 and fractions thereof by means of TLC, HPLC-DAD and LC-MS. SPE-40 contained the main compounds responsible for the antiproliferative activity on HL-60 cells. Thus, a phenolic fraction with high antiproliferative activity on HL-60 cells was obtained from ASMq through the bioassay-guided fractionation process. This could provide a better pharmaceutical formulation that minimizes the administration inconveniencies of a high volume (1.5 L per day) of ASMq decoction for cancer patients.

## 1. Introduction

*Abnormal Savda Munziq* (ASMq) is a medicinal herbal preparation used in the traditional Uighur medicine in Xinjiang region of China [1]. ASMq includes ten medicinal species represented by *Cordia dichotoma* Forst. f.,* Anchusa italica* Retz., *Glycyrrhiza uralensis* Fisch., *Adiantum capillus-veneris* L., *Euphorbia humifusa* Willd., *Ziziphus jujuba* Mill., *Lavandula angustifolia* Mill., *Foeniculum vulgare* Mill., *Melissa officinalis* L., and *Alhagi pseudoalhagi* Desv. The mixture is administered as a decoction and is regularly used for the prevention and treatment of diabetes, cardiovascular diseases, chronic asthma, and cancer. For liver, stomach, and colon cancer, the recommended dose of the decoction is 500 mL three times a day [2].

Recent studies have shown that ASMq can significantly inhibit the growth and viability of Hep G2 human hepatoma cell line [2–4]. ASMq has also been reported to scavenge free radicals [1]. In addition, some species included in ASMq were reported to show *in vitro* inhibitory activity on different tumor cell lines. For example, *G. uralensis* inhibited the growth of Hep3B human hepatoma [3] and MCF-7 breast cancer cell line [4]. *M. officinalis* exerted inhibitory activity against continuous cell culture of HEp-2 cells derived from human laryngeal cancer [5], HeLa, and MCF-7 cell lines [6]. *Z. jujuba* decreased the viability of Hep G2 cell line [7]. Some of these species also showed *in vitro* antiproliferative activities on HL-60 cell line [8].

Although the effect of ASMq and species included in its composition has been recorded on different cancer cells, the high administration volume of the preparation (1.5 L per day) is not only inconvenient for the patients, but also adds with a high percentage to the daily recommended fluid intake. The European Food Safety Authority recommends a drinking volume of around 1.5 L per day. In the USA, The Food and Nutrition Board 2004 states a required amount of fluid intake (water and beverages) of 3 L per day for adult men and 2.2 L per day for adult women [9–11].

 Thus, the consumption of 1.5 L day^−1^ of ASMq would represent around 100% of the required fluid intake as considered by the European forums and around 50% of the American recommended fluid intake. Therefore, reducing the volume of ASMq decoction, while preserving the antiproliferative effect of the mixture, would provide a more convenient modern formulation for the treatment of cancer patients. The present study aimed at obtaining a highly active antiproliferative fraction of ASMq using bioassay-guided fractionation and cell viability evaluation.

## 2. Experimental

### 2.1. Plant Materials

*Pobumuguo* (fruits of* C. dichotoma*), *Niushecao* (whole plant of* A. italica*), *Gancao* (root of *G. uralensis*), *Tiexianjue* (whole plant of* A. capillus-veneris*), *Dijincao* (whole plant of *E. humifusa*), *Hongzao* (fruits of *Z. jujuba*), *Xunyicao* (aerial part of* L. angustifolia*), *Xiaohuixiang* (fruits of *F. vulgare*), *Mifenghua* (whole plant of *M. officinalis*), and *Citang* (sugar secretion from *A. pseudoalhagi*) were purchased from Xinjiang Hospital of Traditional Uighur Medicine, Urumqi, China in September 2007. The plant material used was unprocessed.

### 2.2. Chemicals and Reagents

Luteolin, rosmarinic acid, apigenin 7-O-glucoside and isorhamnetin 3-O-rutinoside were obtained from Extrasynthèse (Genay, France), caffeic acid from Sigma-Aldrich (St. Louis, Mo, USA), and formononetin, rutin, isoquercitrin and gallic acid from Carl Roth (Karlsruhe, Germany). Acetonitrile (HPLC grade) was purchased from VWR International (Leuven, Belgium). Glacial acetic acid (purity > 99.8%) and DMSO were obtained from Carl Roth (Karlsruhe, Germany). Folin-Ciocalteu's phenol reagent was purchased from Merck (Darmstadt, Germany), HPD-300 macroporous resin from Cang Zhou Bonchem Co., Ltd. (Hebei, China), Polyamide resin from Taizhou Luqiao Biochemical Corporation (Zhejiang, China), SPE Mega Bond Elut C-18 cartridges from Varian (Middelburg, The Netherlands). TLC silica gel 60 F_254_ plates were obtained from Merck (Darmstadt, Germany). All other reagents used were of analytical grade.

### 2.3. Preparation and Fractionation

#### 2.3.1. Preparation of ASMq

ASMq was prepared according to a previously reported procedure [12]. The plant material was separately ground and mixed in the following ratio: *C. dichotoma* (10.6), *A. italica* (10.6), *G. uralensis* (7.1), *A. capillus-veneris* (4.9), *E. humifusa* (4.9), *Z. jujuba* (4.9), *L. angustifolia* (4.9), *F. vulgare* (4.9), *M. officinalis* (4.9), and *A. pseudoalhagi* (42.3). The mixture was decocted in boiling water in a ratio of 1 : 10 (w/v) for 3 h. After filtration, the residue was reextracted for 3 h, two times in the same volume of boiling water. The resulting crude extract was filtered, evaporated to dryness under reduced pressure, and pulverized. The obtained powder was used for this study. The yield was 39.9% (w/w) with respect to the total mass of dry materials.

#### 2.3.2. Preparation of Different Extracts of ASMq

350 g dry extract of ASMq was dissolved in 1050 mL hot H_2_O (60°C), filtered, and cooled to room temperature. The resulting solution was passed through a column (125 cm × 5 cm) filled with HPD-300 macroporous adsorbent resin. The column was eluted with 3 bed volumes (BV) of H_2_O dest. The H_2_O eluate was concentrated, and then 95% EtOH was added to obtain a final concentration of 70% EtOH. The mixture was stirred for 10 min. The resulting precipitate was collected and redissolved in H_2_O and the EtOH precipitation was repeated once again. The resulting precipitate was decolored with activated carbon and evaporated under reduced pressure to obtain extract 1 (Extr-1, 54.9 g). The HPD-300 macroporous column was sequentially eluted with 3 BV of 20% EtOH and 3 BV of 60% EtOH, and the resulting eluates were collected separately and evaporated under reduced pressure to dryness to obtain extract 2 (Extr-2, 15.7 g) and extract 3 (Extr-3, 25.3 g), respectively. The yields (w/w) of Extr-1, Extr-2, and Extr-3 were 15.69%, 4.49%, and 7.23% of the dry weight of ASMq, respectively.

#### 2.3.3. Preparation of Extract 4

Dry extract of ASMq (350 g) was dissolved in 1050 mL hot H_2_O (60°C) and filtered. 1.5 volumes of 95% EtOH were added; the mixture was stirred for 20 min and then allowed to stand for 24 h. The resulting supernatant layer was filtered, concentrated under reduced pressure to 350 mL and subjected to column chromatography (125 cm × 5 cm) on polyamide resin. The column was eluted first with H_2_O dest. (3 BV), followed by 60% EtOH (3 BV). The fraction eluted with H_2_O dest. was discarded, while the fraction eluted with 60% EtOH was evaporated under reduced pressure to dryness to obtain extract 4 (Extr-4, 12.2 g). The yield (w/w) of Extr-4 was 3.49% with respect to the dry weight of ASMq.

#### 2.3.4. Fractionation of Extr-4

Extr-4 was further fractionated by solid phase extraction (SPE). 50 mg of Extr-4 were dissolved in 1 mL MeOH 40% and applied onto an SPE column preconditioned with 2 reservoir volumes (RV) MeOH and 2 RV H_2_O. The cartridge was sequentially eluted with 2 RV of H_2_O and 20%, 40%, 60%, and 80% MeOH at a flow rate of 1 mL min^−1^. The resulting eluates were collected separately and evaporated under reduced pressure to yield SPE-0 (1.1 mg), SPE-20 (1.0 mg), SPE-40 (11.7 mg), SPE-60 (11.9 mg) and SPE-80 (5.2 mg), respectively. The yields (w/w) of these fractions were 0.15% (SPE-0), 0.14% (SPE-20), 1.63% (SPE-40), 1.66% (SPE-60), and 0.73% (SPE-80) of the dry weight of ASMq, respectively.

**Figure 1:**
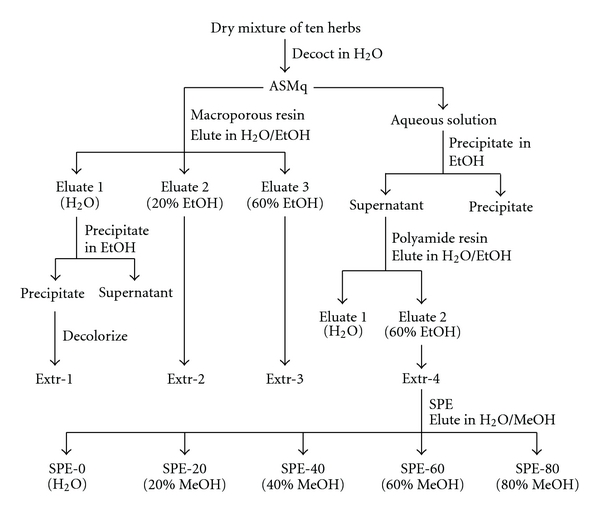
A flow chart of the ASMq preparation and fractionation procedure.

A flow chart of the ASMq preparation and fractionation procedure is shown in Figure 1.

### 2.4. Cell Culture

The HL-60 human promyelocytic leukemia cell line was obtained from the American Type Culture Collection (ATCC). Cell medium RPMI 1640 and its supplements were obtained from Life Technologies, Inc., USA. The HL-60 cells were routinely cultured in RPMI 1640 medium, supplemented with 10% (v/v) heat inactivated foetal bovine serum, 1% L-glutamine, and 1% penicillin/streptomycin and grown in a humidified atmosphere with 5% CO_2_ at 37°C.

### 2.5. Evaluation of Cell Viability

Cells were seeded at a density of 0.1 × 10^6^ cells mL^−1^ in 12-well plates and grown for 24 h. Cells were then incubated with 100 *μ*g mL^−1^ and 250 *μ*g mL^−1^ ASMq, extracts (Extr-1, Extr-2, Extr-3, and Extr-4) and 50 *μ*g mL^−1^ SPE fractions (SPE-0, SPE-20, SPE-40, SPE-60, and SPE-80), respectively. In order to determine the IC_50_ value of Extr-4, cells were exposed to increasing concentrations (0, 25, 50, 100, 200, and 250 *μ*g mL^−1^) of Extr-4. Prior to use, the stock solutions of all dry extracts (ASMq extracts and SPE fractions) were prepared in 60% EtOH and then filtered through 0.20 *μ*m sterile syringe filters. The final concentration of EtOH in culture medium during the treatment of cells did not exceed 0.5% (v/v). Vehicle-treated cells (0.5% EtOH) were tested as control samples. After 24 h, 48 h, and 72 h of incubation, the cells were stained with tryptan blue (0.4%) and counted with a cell counter (Vi-CELL XR Cell Viability Analyzer, Beckman, USA). The cell viability was expressed as the percentage of control. Analyses were performed in triplicate, values are given as mean ± SD.

### 2.6. TLC Analysis

TLC analyses of ASMq extracts and SPE fractions were performed on silica gel 60 F_254_ plates. Extr-1, Extr-2, Extr-3, and Extr-4 were dissolved in H_2_O, 20% EtOH, 40% EtOH, and 60% EtOH, respectively (20 mg mL^−1^), and 10 *μ*L aliquots were applied to a TLC plate. As mobile phase CHCl_3_/MeOH/H_2_O (70 : 22 : 3.5, v/v/v) was used, the plate was sprayed with 0.5% fast blue salt B aqueous solution [13]. Evaluation was done under visible light. Rosmarinic acid was used as reference compound. 

SPE fractions were dissolved in 60% MeOH (20 mg mL^−1^) and 5 *μ*L aliquots were applied to a TLC plate and developed with EtOAc/HCOOH/HAc/H_2_O (100 : 11 : 11 : 26, v/v/v/v). The plate was then sprayed with natural products spraying reagent (1% methanolic diphenylboric acid-*β*-ethylamino ester) followed by 5% ethanolic polyethylene glycol-4000 [13]. The plate was investigated under 365 nm. Rutin, hyperoside, and astragalin were used as reference compounds.

### 2.7. HPLC-DAD and LC-MS Analysis

HPLC-DAD analyse was conducted on a Shimadzu LC-10AD liquid chromatograph equipped with a SCL-10A system operator, a SPD-M20A diode array detector, a SIL-10AD auto injector and a DGU-14A degasser. The LC-MS analyses were performed on an UltiMate 3000 RSLC-series system (Dionex, Germering, Germany) coupled to a 3D quadrupole ion trap mass spectrometer equipped with an orthogonal ESI source (HCT, Bruker Daltonics, Bremen, Germany). HPLC separation was carried out on a Hypersil BDS-C18 column (4 × 250 mm, 5 *μ*m, Thermo Scientific, Waltham, Mass, USA) at a flow rate of 1.2 mL min^−1^. Water (pH 2.8 with acetic acid) and MeCN (with the same amount of acetic acid) were used as mobile phase A and B, respectively. The following gradient program was used: 15% B (0 min), 41.3% B (45 min), 95% B (46 min), and 95% B (51 min). The eluent flow was split roughly 1 : 8 before the ESI ion source, which was operated as follows: capillary voltage: 3.7 kV, nebulizer: 30 psi (N_2_), dry gas flow: 8 L min^−1^ (N_2_), and dry temperature: 340°C. The mass spectrometer was operated in an automated data-dependent acquisition (DDA) mode where each MS scan (*m/z* 80–1100, average of 5 spectra) was followed by MS^2^ scans (*m/z* 40–1100, average of 3 spectra, isolation window of 4 Th, fragmentation amplitude of 1.0 V) of the two most intense precursor ions, and MS^3^ scans (*m/z* 40–1100, average of 3 spectra, isolation window of 4 Th, fragmentation amplitude of 1.0 V) of the most intense fragment ion in each MS^2^ scan. In additional LC-MS experiments, the instrument was operated in alternating ion MS^1^ mode. 

The sample injection volume was 10 *μ*L. 2 mg of Extr-4 as well as 2 mg of each of the SPE fractions were separately sonicated with 1 mL of 60% MeOH-DMSO (4 : 1, v/v) for 5 min at room temperature. Standard phenolic compounds were dissolved in MeOH. Identification of compounds was achieved by comparing retention times as well as UV and MS^n^ spectra.

### 2.8. Total Flavonoid Content

The total flavonoid content was determined by aluminium nitrate method [14]. 30 mg of ASMq and 3 mg of Extr-4 were dissolved in 1 mL H_2_O and 1 mL of 60% EtOH in a 5 mL volumetric flask, respectively. 2 mL of H_2_O and 0.15 mL of aqueous 5% NaNO_2_ solution were added, followed by 0.15 mL of 10% Al(NO_3_)_3_ 6 min later. The mixture was allowed to stand for another 6 min at room temperature. Then, 1 mL of 1 M NaOH was added. The sample was immediately diluted with H_2_O to a final volume of 5 mL and mixed. A blank without sample solution was prepared in parallel. Rutin was used as standard to establish the calibration curve. Absorbances of samples were determined at 510 nm on a spectrophotometer (Beckman DU-640). Total flavonoid content was expressed as mg rutin equivalent (RUE) g^−1^ dried extract. Data for ASMq and Extr-4 were reported as mean ± SD for five replicates and for triplicates, respectively.

### 2.9. Total Phenolic Content

The Folin-Ciocalteu method was used to determine the total phenolic content [15]. 160 *μ*L of sample (6 mg ASMq dissolved in 1 mL H_2_O and 0.6 mg Extr-4 dissolved in 1 mL 60% EtOH, respectively) were added to a 5 mL volumetric flask and diluted with H_2_O dest. to 2.5 mL. 0.25 mL of Folin-Ciocalteu reagent were added. 6 min later, 0.75 mL of 20% aqueous Na_2_CO_3_ solution were added, diluted with H_2_O to a final volume of 5 mL and mixed. After incubation at room temperature for 2 h, the absorbance of the reaction mixture was measured at 765 nm against a blank containing only solvent and reagents. Gallic acid was used as standard for the calibration curve. Total phenolic content was expressed as mg gallic acid equivalent (GAE) g^−1^ dried extract. Data for ASMq and Extr-4 were reported as a mean ± SD for five replicates and for triplicates, respectively.

### 2.10. Statistical Analysis

The results of cell viability were presented as means ± SD. Statistical comparison was performed using the one-way analysis of variance (ANOVA) followed by Tukey's test (in case of equal variance) or Dunnett's T3 test (in case of unequal variance). These tests were performed using SPSS 13 for Windows. *P* < 0.05 was considered statistically significant. IC_50_ values and their 95% confidence limits were estimated by a sigmoidal dose-response model with variable slope (GraphPad Prism software, version 4.03).

## 3. Results and Discussion

**Figure 2:**
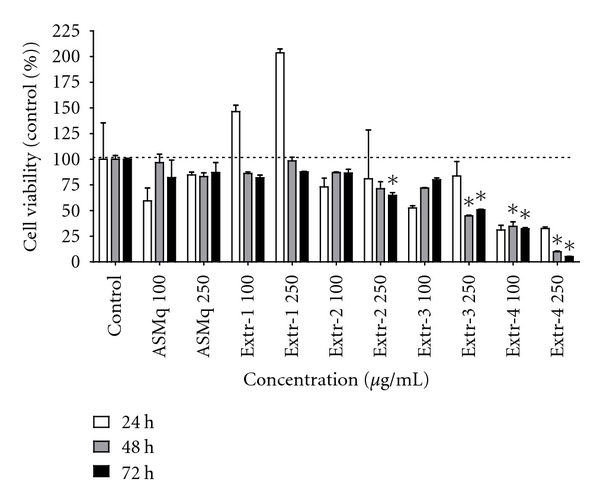
Effect of ASMq, Extr-1, Extr-2, Extr-3, and Extr-4 on the viability of HL-60 cells. Cells were seeded in 12-well plates, grown for 24 h and then treated with 100 *μ*g/mL and 250 *μ*g/mL ASMq, Extr-1, Extr-2, Extr-3, and Extr-4. After 24 h, 48 h, and 72 h cell viability was assessed by tryptan blue exclusion method. Data represent the percentage of control value and are expressed as mean ± SD of triplicate cultures. *P < 0.05, as compared to control cells.

**Figure 3:**
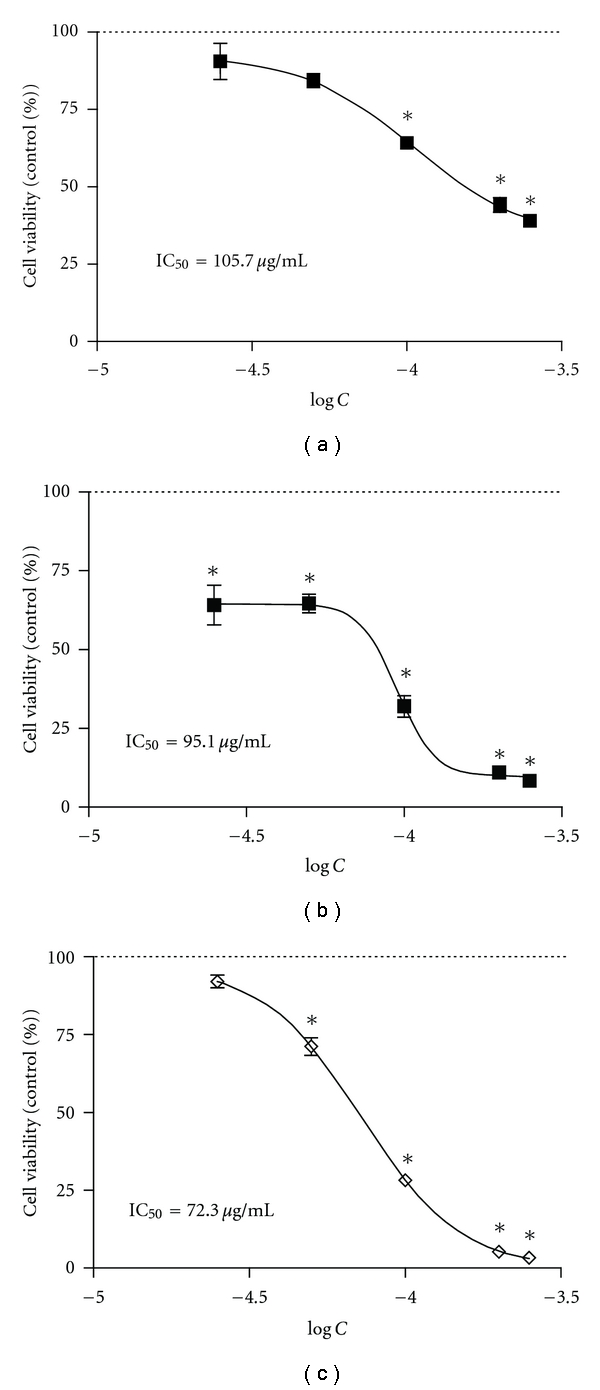
Dose-response curves of antiproliferative activity of Extr-4 after 24 h (a), 48 h (b), and 72 h (c). HL-60 cells were cultured in 12-well plates for 24 h to reach logarithmic growth phase and then incubated with 25–250 *μ*g/mL Extr-4. Cell viability was estimated by tryptan blue staining and cell counting after 24 h, 48 h, and 72 h. Data are presented as percentage of control value. The horizontal axis represents a log scale of concentration of Extr-4. The IC_50_ was calculated using a sigmoidal dose-response model with variable slope. The curve represents the average of experiments performed in triplicate. *P < 0.05, compared with respective control value (24 h : 100.00 ± 8.50%; 48 h : 100.00 ± 8.84%, 72 h : 100.00 ± 22.26%).

In the first step of this study, three extracts, Extr-1, Extr-2, and Extr-3, were obtained from ASMq by macroporous resin chromatography and tested for their effect on cell viability.  Figure 2 shows the activity of ASMq and the corresponding extracts on HL-60 cell viability. ASMq and Extr-1 (250 *μ*g mL^−1^) showed no remarkable cytotoxicity after 72 h, reducing cell viability to 87.1 ± 16.7% and 87.7 ± 1.3%, respectively. Extr-1 enhanced cell proliferation after 24 h for both tested concentrations (100 and 250 *μ*g mL^−1^). ASMq was previously reported to contain 58% saccharides as glucose equivalents [16]. Therefore, the enhanced cell proliferation of HL-60 cells after 24 h in the presence of Extr-1 could be due to the high content of saccharides in these extracts. Unlike ASMq and Extr-1, Extr-2 (250 *μ*g mL^−1^) showed higher cytotoxicity, inducing a decline in cell viability to 64.8 ± 4.8%, while Ext-3 (250 *μ*g mL^−1^) exhibited the highest cytotoxicity, decreasing cell viability to 50.83 ± 0.70% after 72 h. Thus, ASMq showed no remarkable activity on HL-60 cell proliferation at the given concentration, while different effects on cell viability were distributed between the three extracts obtained from ASMq, with the most apolar of the extracts showing increased antiproliferative effect. The low activity of ASMq is in line with the high dose of ASMq decoction (1.5 L per day) required for the treatment of cancer patients. The result of TLC analysis revealed that phenolic compounds were absent in Extr-1 but could be detected in Ext-2 and Extr-3. When compared to Extr-2, Extr-3 showed an enriched phenolic fingerprint. This implied a possible correlation between the phenolic compounds and the cytotoxic activity on HL-60 cells. Therefore, a polyamide chromatography was performed on ASMq to obtain an extract enriched in phenolic compounds. The resulting extract, Extr-4, showed higher cytotoxicity than Extr-2 and Extr-3, reducing cell viability to 5.29 ± 0.28% at a concentration of 250 *μ*g mL^−1^, after 72 h (Figure 2).  Figure 3 shows the dose-response curve for Extr-4 when tested on HL-60 cells after 24 h, 48 h, and 72 h. The IC_50_ values were 105.7 *μ*g mL^−1^ (24 h), 95.1 *μ*g mL^−1^ (48 h) and 72.3 *μ*g mL^−1^ (72 h).

According to preliminary TLC analysis, the number of phenolic compounds increased from Extr-1 to Extr-4 with the majority present in the latter. The total flavonoid content and total phenolic content of ASMq were 25 ± 1 mg RUE g^−1^ dried extract and 38 ± 1 mg GAE g^−1^ dried extract, respectively, whereas those of Extr-4 were 221 ± 9 mg RUE g^−1^ dried extract and 333 ± 7 mg GAE g^−1^ dried extract, respectively. Both the flavonoid content and the phenolic content increased to approximately 9-fold in Extr-4 versus ASMq. These results confirmed that the phenolic compounds were enriched in Extr-4 through polyamide resin chromatography.

**Figure 4:**
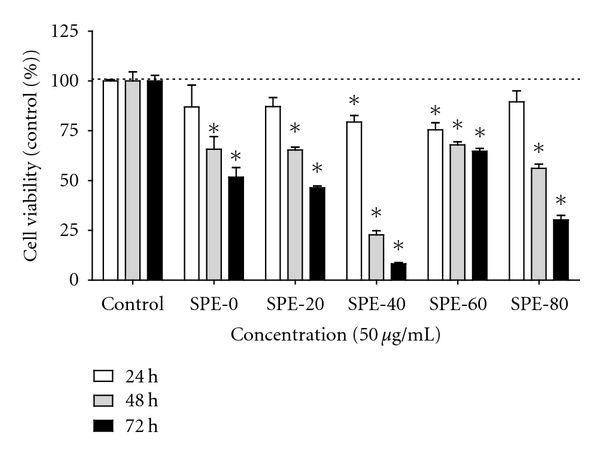
Viability of HL-60 cells after 24 h, 48 h, and 72 h of treatment with SPE-0, SPE-20, SPE-40, SPE-60, and SPE-80 subfractions. Cell viability was assessed by tryptan blue exclusion method and is presented as percentage of control value. Data are expressed as mean ± SD of two independent experiments, each of which was done in triplicate. *P < 0.05, compared with control. Each sample was tested at a concentration of 50 *μ*g/mL.

Using SPE, Extr-4 was further fractionated into five fractions, SPE-0, SPE-20, SPE-40, SPE-60, and SPE-80. According to cell viability assessment after 72 h, all fractions showed various degrees of cytotoxicity on HL-60 cells (Figure 4). SPE-40 (50 *μ*g mL^−1^) was found to exhibit the strongest inhibitory effect on the viability of HL-60 cells by inducing a time-dependent decrease in cell viability to 8.2 ± 1.5%. The TLC analyses revealed the presence of flavonoids in all SPE fractions except SPE-80. The majority of flavonoids were present in SPE-40, implying that flavonoids may be the most active phenolic compounds in SPE-40.

**Figure 5:**
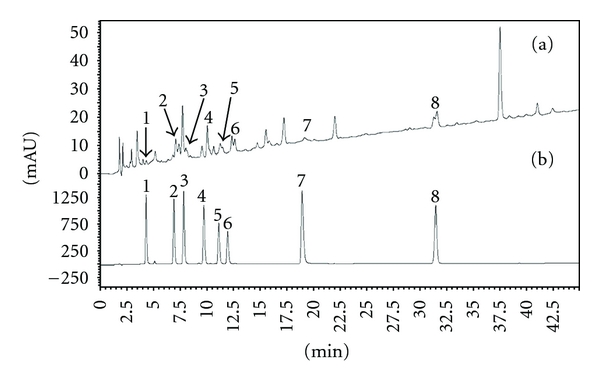
Reversed phase HPLC chromatograms of standard phenolic compounds (a) and Extr-4 (b) at a wavelength of 254 nm. (**1**) caffeic acid, (**2**) rutin, (**3**) isoquercitrin, (**4**) isorhamnetin 3-O-rutinoside, (**5**) apigenin 7-O-glucoside, (**6**) rosmarinic acid, (**7**) luteolin, and (**8**) formononetin.

**Figure 6:**
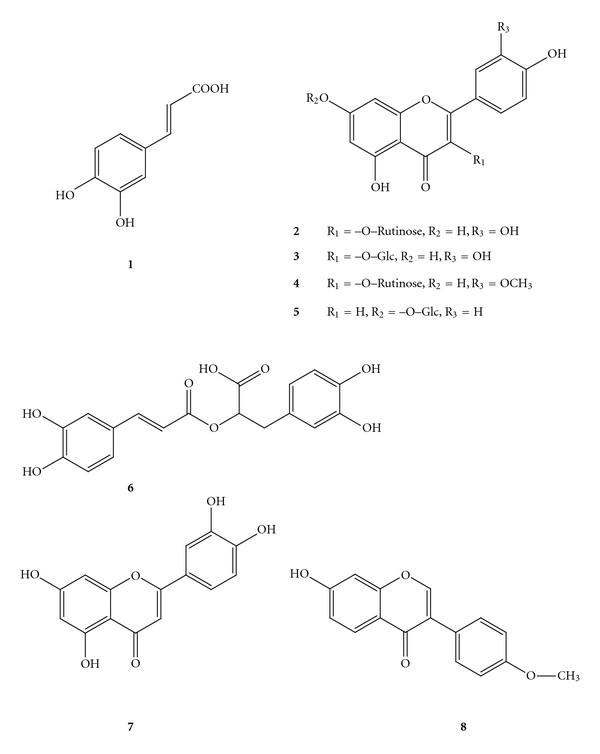
Compounds identified in Extr-4 and its fractions: (**1**) caffeic acid, (**2**) rutin, (**3**) isoquercitrin, (**4**) isorhamnetin 3-O-rutinoside, (**5**) apigenin 7-O-glucoside, (**6**) rosmarinic acid, (**7**) luteolin, and (**8**) formononetin.

**Figure 7:**
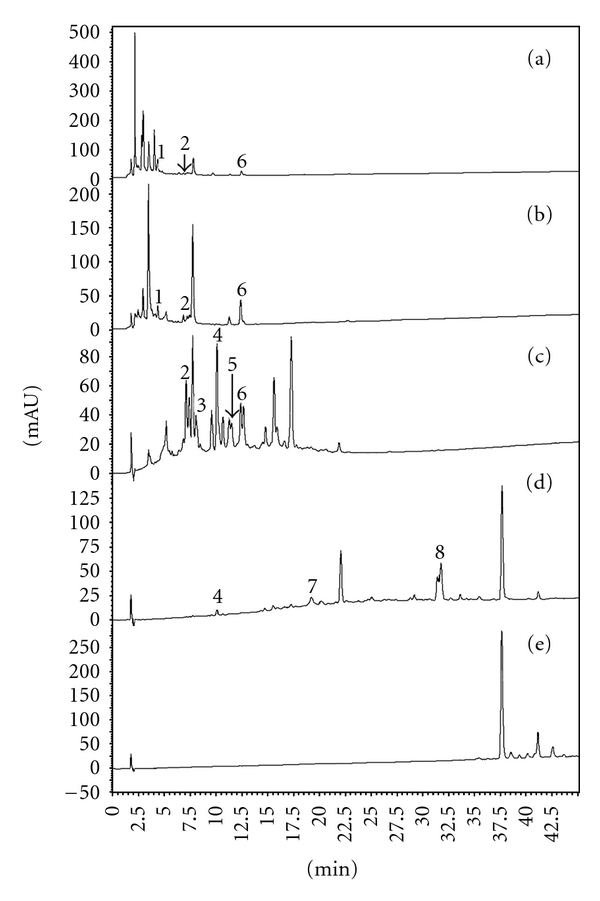
HPLC chromatograms of five SPE fractions at a wavelength of 254 nm. (a) SPE-0, (b) SPE-20, (c) SPE-40, (d) SPE-60, (e) SPE-80, (**1**) caffeic acid, (**2**) rutin, (**3**) isoquercitrin, (**4**) isorhamnetin 3-O-rutinoside, (**5**) apigenin 7-O-glucoside, (**6**) rosmarinic acid, (**7**) luteolin, and (**8**) formononetin.

The identification of phenolic compounds in Extr-4 and SPE fractions was performed using HPLC-DAD and LC-MS. The HPLC chromatogram of Extr-4 recorded at 254 nm is shown in Figure 5. Eight phenolic compounds, namely, caffeic acid (**1**), rutin (**2**), isoquercitrin (**3**), isorhamnetin 3-O-rutinoside (**4**), apigenin 7-O-glucoside (**5**), rosmarinic acid (**6**), luteolin (**7**), and formononetin (**8**), were identified in Extr-4 by comparison of their retention time, and UV and MS spectral characteristics to those of standards in Figure 5(a), as well as by spiking the sample with standards. The chemical structures of compounds **1** *∼* **8** are shown in Figure 6. Several phenolic compounds that have been described as chemical constituents of the species included in ASMq, such as chlorogenic acid, luteolin 3′,7-O-diglucoside, ferulic acid, eriodictyol 7-O-glucoside, narirutin, rhamnetin, rhoifolin, and isovitexin, were not found in Extr-4. Some of the phenolic compounds we identified in Extr-4 by LC-MS were also found in SPE-0, SPE-20, SPE-40, and SPE-60. For example, **1**, **2,** and **6** were identified in SPE-0 (Figure 7(a)) and SPE-20 (Figure 7(b)) in varying ratios. Five phenolic compounds, **2**–**6**, were identified in SPE-40 (Figure 7(c)), whereas **4**, **7,** and **8** were found in SPE-60 (Figure 7(d)). Compound **3** was identified only in SPE-40. Based on the results of TLC and HPLC analyses, SPE-80 may not contain phenolic compounds (Figure 7(e)) but is mainly enriched in licorice triterpene saponins (e.g., glycyrrhizic acid) according to tentative identification by LC-MS (data not shown).

Phenolic compounds are well studied secondary plant metabolites that have been thoroughly investigated due to their presence in the human diet and their biological properties, including their beneficial effects on health. One of these effects is their capacity to interfere with all stages of the cancer process [17, 18]. Several flavonoids have been shown to suppress carcinogenesis in various animal models [19]. A growing number of epidemiological studies suggest that high flavonoid intake may be correlated with decreased cancer risk [20–22].

The phenolic compounds detected in Extr-4 and its SPE fractions were also previously identified in species from the composition of ASMq. For example, **1** was identified in *L. angustifolia* [23] and *M. officinalis* [24], **2** in *Z. jujuba* [25], *A. capillus-veneris *[26],* C. dichotoma *[27], *G. uralensis* [28], and *F. vulgare* [29], and **3** in *A. capillus-veneris* [26], *G. uralensis *[28], and *M. officinalis* [30]. **4** was found in *C. dichotoma *[27], *G. uralensis *[28], and* F. vulgare* [29] and **5** in *E. humifusa *[31] and *M. officinalis *[30]. **6** was identified in *C. dichotoma *[27],* F. vulgare* [29], and* M. officinalis *[24]. **7** and **8** were found in *M. officinalis *[32] and *G. uralensis *[33], respectively. 

Compounds **2** [34] and **8** [35] were reported to possess no remarkable cytotoxicity on HL-60 cells. **4** showed no distinctive cytotoxicity on HT-29 human colon carcinoma, MCF-7 human breast carcinoma, and Hep G2 human hepatoma cell lines [36]. **3** showed antiproliferative effects on B16F10 murine melanoma [37] and MCF-7 human breast carcinoma cell lines [38]. **5** possessed moderate cytotoxic effects on human prostate cancer cell lines derived from different metastatic sites, such as LNCaP, DU145, and PC-3 cells [39]. **1** [40] and **6** [41] showed low cytotoxic effects on HL-60 cell line, while 7 [42] exhibited a strong cytotoxic activity on HL-60 cells. Thus, the phenolic compounds identified in Extr-4 have been previously shown to have no remarkable (**2**, **4**, **8**), moderate (**1**, **3**, **5**, **6**), or strong (**7**) cytotoxic effect on different cancer cell lines. 

SPE-0 and SPE-20 contain compounds that have been described as moderate cytotoxic which can explain their observed activity in the viability tests. The concentration of **7**, a strong cytotoxic compound, in SPE-60 was low as shown by the HPLC analysis, justifying the moderate effect of this fraction. The characterization of SPE-40, the most active fraction, identified compounds described as moderate (**3**, **5**, **6**) or very low cytotoxic (**2**, **4**), suggesting that **3**, **5,** and **6** could, at least in part, contribute to the antiproliferative activity of SPE-40 on HL-60 cells. The mechanisms of antiproliferative effects of these compounds were investigated in some studies. For example, **3** was reported to reduce glioblastoma cell growth without inducing apoptosis, possibly by modulating the control of the cell cycle. It was also suggested that *β*-catenin-mediated signaling may be involved on the antiproliferative activity of **3** [43]. **5** inhibited cancer cell growth through deconjugation of glycosides that occurs in the cellular compartment to produce aglycone and apigenin [44]. Apigenin itself exhibits a significant growth inhibition against hepatoma cell lines and induces apoptosis in Hep G2 cells [45]. Its apoptotic mechanism might be mediated through the p53-dependent pathway and the induction of p21 expression [45] or through a mitochondria/caspase-pathway [46]. It was also reported that apigenin inhibits cancer cell proliferation through G2/M cell cycle arrest [47] and Er*β* [48]. The authors have found no report on the mechanism underlining the antiproliferative activity of **6** against cancer cells. 

However, these results also imply that the identified phenolics are not the only compounds responsible for the antiproliferative activity of SPE-40 and suggest the possible presence of one or more compounds with stronger activity. The latter could contribute to the cytotoxicity of the identified phenolic compounds by both additive and synergistic effects. The majority of the uncharacterized compounds in SPE-40 showed UV-Vis spectral characteristics similar to flavanones, chalcones, and ellagic acid derivatives (data not shown), which were reported to possess strong antiproliferative activities on different cancer cells [49–52]. This could also be confirmed by LC-MS, which indicates the presence of several typical liquorice flavanones (e.g., liquiritin apioside, liquiritin, and liquiritigenin) and chalcones (e.g., isoliquiritin apioside and isoliquiritin) based on the comparison of multistage MS data with published results (data not shown) [53], indicating that phenolic compounds, with possible strong antiproliferative activity, constitute most of the remaining uncharacterized compounds in SPE-40.

## 4. Conclusion

In summary, our study focused on the bioassay-guided fractionation of ASMq aiming at the reduction of the administration volume of the ASMq decoction (1.5 L day^−1^) and the modernization of its pharmaceutical formulation. Four extracts, namely, Extr-1, Extr-2, Extr-3, and Extr-4, were prepared from ASMq. Extr-4 exerted the strongest antiprolifertive activity on HL-60 cells. Extr-4 was further fractionated into five fractions by solid phase extraction, of which SPE-40 showed the strongest inhibitory effect on HL-60 cells. The results of the cell viability assay, quantification assay, and the TLC and LC-MS analyses on Extr-4 and the SPE fractions revealed that the main bioactive components with antiproliferative activity against HL-60 cell line could be phenolic compounds. The substances identified in Extr-4 and its SPE fractions were caffeic acid, rutin, isoquercitrin, isorhamnetin 3-O-rutinoside, apigenin 7-O-glucoside, rosmarinic acid, luteolin, and formononetin. Isoquercitrin, apigenin 7-O-glucoside, and rosmarinic acid may contribute to the cytotoxicity of SPE-40 on HL-60 cells. The results also suggest that other compounds than the identified phenolics add to the antiproliferative effect of ASMq. However, fraction SPE-40 obtained in the course of our work could significantly reduce the high therapeutic dosage of ASMq (500 mL three times a day). One dose of ASMq was reduced from 500 mL (~200 g dry weight) to 1.6 g fraction SPE-40. Thus, the bioassay-guided fractionation of ASMq could lead to the enrichment of active compounds in an active fraction and to the diminution of the administration dose of the TCM preparation, providing a better pharmaceutical formulation for cancer patients.
